# A Retrospective Analysis of Characteristics Favouring In-Hospital Resuscitation Plan Completion, Their Timing, and Associated Outcomes

**DOI:** 10.3390/jcm13144098

**Published:** 2024-07-13

**Authors:** Sara L. Schaefer, Campbell H. Thompson, Samuel Gluck, Andrew E. C. Booth, Colette M. Dignam

**Affiliations:** 1 Adelaide Medical School, Faculty of Health and Medical Sciences, University of Adelaide, 4 North Terrace, Adelaide, SA 5000, Australia; 2 Central Adelaide Local Health Network, Adelaide, SA 5000, Australia; 3 Northern Adelaide Local Health Network, Adelaide, SA 5092, Australia

**Keywords:** inpatient resuscitation orders, advance care resuscitation planning, POLST, not for CPR, goals of care, end of life

## Abstract

**Background:** Comprehensive resuscitation plans document treatment recommendations, such as ‘Not for cardiopulmonary resuscitation’. When created early in admission as a shared decision-making process, these plans support patient autonomy and guide future treatment. The characteristics of patients who have resuscitation plans documented, their timing, and associations with clinical outcomes remain unclear. **Objectives:** To characterise factors associated with resuscitation plan completion, early completion, and differences in mortality rates and Intensive Care Unit (ICU) admissions based on resuscitation plan status. **Methods**: This retrospective study analysed non-elective admissions to an Australian tertiary centre from January to June 2021, examining plan completion timing (early < 48 h, late > 48 h) and associations with mortality and ICU admission. **Results**: Of 13,718 admissions, 5745 (42%) had a resuscitation plan recorded. Most plans (89%) were completed early. Furthermore, 9% of patients died during admission, and 8.2% were admitted to the ICU. For those without resuscitation plans, 0.5% died (*p* < 0.001), and 9.7% were admitted to the ICU (*p* = 0.002). Factors associated with plan completion included a medical unit, in-hours admission, older age, female gender, limited English proficiency, and non-Indigenous status. Plans completed late (>48 h) correlated with a higher mortality (14% vs. 9%; *p* < 0.001) and more ICU admissions (25% vs. 6%; *p* < 0.001). Aboriginal and/or Torres Strait Islander patients were often overlooked for resuscitation documentation before death. No resuscitation plans were documented for 62% of ICU admissions. **Conclusions**: Important disparities exist in resuscitation plan completion rates across highly relevant inpatient and demographic groups.

## 1. Introduction and Background

International and local guidelines support early and recurrent anticipatory goals-of-care discussions for patients who are dying or medically unstable, or who face a critical illness or complication [1,2,3,4]. In South Australia, the standardised electronic 7-Step Pathway resuscitation plan (Additional File S1 in Supplementary Materials) serves as the primary document for resuscitation planning in all public tertiary centres, including decisions about goals of care and cardiopulmonary resuscitation (CPR). After discussions between a clinician and the patient or their substitute decision-maker, decisions about future care are recorded using a template featuring tick-box options (such as ‘No CPR’, ‘Not for intubation’, or ‘For full resuscitation’) and a free-text box available to document alternative care directives including a palliative approach. Clinicians may consider initiating a resuscitation plan based on triggers including objective clinical deterioration, meeting indicators from the Supportive and Palliative Care Indicators Tool [5], or the reasonable likelihood of mortality within 12 months. The triggers for implementing a resuscitation plan and the format of the 7-Step resuscitation plan share many characteristics with similar processes in place around the world [6,7].

Resuscitation plans documented in an electronic medical record provide succinct and readily accessible information about patients’ resuscitation preferences during acute deterioration. The early completion of a resuscitation plan (defined as within 48 h of unplanned hospital admission) offers several benefits. It allows meaningful patient involvement before they become too unwell or confused to participate actively [3], and removes a potentially emergent decision-making burden for a distressed surrogate [8,9]. Furthermore, early resuscitation plans reduce the use of unwanted, costly, and burdensome interventions for patients, and reduce the loss of dignity and suffering for the patient and their family at the end of life [10,11,12]. For patients who survive to discharge or do not require an Intensive Care Unit (ICU) admission, a resuscitation plan serves as an opportunity to honour patient autonomy, individualise their journey, and minimise harmful or non-beneficial interventions in the future.

At this tertiary centre, replacing stand-alone No-CPR orders with comprehensive plans resulted in a marked increase in documented resuscitation planning. In 2017, documentation was completed in the first 48 h in two-thirds of Australian general medicine inpatients aged over 70 years; double the rates for similar patients admitted in 2011 [13]. Despite this widespread adoption [13] and guidelines recommending early documentation [1,2,3,4], patient demographics for early resuscitation planning in the Australian inpatient population remain unknown. Additionally, in terms of understanding the disparities in outcomes, particularly ICU admission and mortality rates among patients with early, delayed or no resuscitation plans necessitates further investigation.

## 2. Objectives

The study had two primary objectives. The first is to identify patient groups most likely to have a resuscitation plan or not during their admission using demographic and hospital data. This objective was further refined by comparing characteristics of patients with an early resuscitation plan to those patients for whom documentation was completed after 48 h of admission (referred to as a delayed resuscitation plan). By separately analysing patient characteristics in relation to mortality rates, we aimed to ascertain if any disparities existed between respective groups and if this aligned with the presence or absence of resuscitation planning.

The second objective was to establish differences in the timing and completion rates of resuscitation plans between those who died or those who were admitted to the ICU. By examining mortality and ICU admission rates across these categories, we sought to provide insights into the impact of timely resuscitation planning on patient outcomes.

## 3. Methodology

### 3.1. Design

This retrospective study was conducted at a South Australian adult tertiary care centre with approximately 800 inpatient beds. Data were extracted from de-identified electronic patient records for individuals admitted to an inpatient service through the emergency department between 1 January 2021 and 30 June 2021. Elective admissions, day-stay patients (defined as stay <24 h), and mental health admissions were excluded from the study. Four “unknown” or “intersex” patients were also excluded in recruitment.

### 3.2. Outcome Measures

Patients were categorised into those with and without a documented resuscitation plan. Patients with a resuscitation plan were then further analysed based on the timing of their documented discussions. The content of individual plans was not analysed. Outcome measures included presence and timing of a completed resuscitation plan, inpatient mortality, and ICU admission.

Demographic data collected included age, gender, primary spoken language, Aboriginal and/or Torres Strait Islander (hereafter, respectfully, Indigenous Australian) status, admitting clinical unit, and time of admission. Medical and surgical patients were defined based on the admitting specialty service. In instances of intra-hospital transfer, the final admitting unit was used. Age was categorised as less than seventy, or seventy years and over. Timing of admission was analysed as in-hours (08:00–16:59 h) compared with out-of-hours (17:00–07:59 h). English-proficient patients were compared with patients with limited English proficiency (LEP)—non-English speakers, those with an unknown documented primary language, and those with English as a second language. Indigenous Australians were compared to non-Indigenous patients; 569 patients who did not state their heritage were excluded from analysis for this variable only. Gender comparison was between male and female. ICU admission was compared between patients with recorded time spent in the ICU versus zero hours in the ICU; 116 patients with no recorded ICU length of stay were excluded from the analysis of ICU outcomes.

### 3.3. Statistical Analysis

Binary outcomes were used for all demographic data to maximise statistical power. Chi-square tests were employed to compare binary data outcomes, with a *p*-value < 0.05 indicating probable significance and a *p*-value < 0.001 indicating statistical significance.

### 3.4. Ethics

Prior to the commencement of this project, it was reviewed by the Human Research Ethics Committee at Central Adelaide Local Health Network (CALHN) Research Services. This service confirmed that no triggers for ethical review were present. No triggers for ethical review were present and individual consent was not obtained given the retrospective nature of the study.

## 4. Results

Over the six-month study period, there were a total of 13,718 patients admitted from the emergency department. Resuscitation plans were completed for 5745 (42%) of these patients. Of this group with documented resuscitation plans, 5107 (89%) plans were completed early in admission. Below, a breakdown of all admissions and those who died is provided [Table 1].

Several factors were significantly associated with resuscitation plan completion. These included admission under a medical unit, female gender, age over 70 years, having LEP, and non-Indigenous status (all *p* < 0.00001). Admissions in-hours was probably a significant factor for increased plan completion rates (*p* = 0.003). Early resuscitation plan completion was significantly associated with admission under a medical team (*p* < 0.00001), and trends were observed for in-hours admission (*p* = 0.026), age over 70 years (*p* = 0.0027), female gender (*p* = 0.039), and non-Indigenous status (*p* = 0.0014). English-speaking proficiency did not affect the timing of completion.

### 4.1. Mortality Outcomes and Resuscitation Plan Status

Risk of death was significantly higher for patients admitted under a medical team, aged over 70 years (both *p* < 0.00001), and those who had LEP (*p* = 0.000026). The risk of death was also higher for patients admitted out-of-hours (*p* = 0.023) with no significant difference between genders or Indigenous Australian status. Notably, 497 (92%) of the 540 patients who died had a documented resuscitation plan before death.

Among the 5745 patients with a documented plan, 497 (9%) died during their admission, compared to 43 (0.54%) of 7973 patients without a resuscitation plan (*p* < 0.00001). There were 5248 (91%) patients with a resuscitation plan that survived to discharge. Death without a resuscitation plan was more likely for patients admitted under a surgical unit, patients aged less than 70 years, and patients identifying as Indigenous Australian (all *p* < 0.00001). Strikingly, 33% of Indigenous Australian deaths occurred with no resuscitation plan in place compared to only 6% in non-Indigenous patient deaths.

Of patients with an early resuscitation plan, 9% died during their admission compared with 14.4% of those with late plans (*p* < 0.00001). Among patients who died, those with early resuscitation plans were more likely to be admitted under medicine (*p* = 0.0021), be female (*p* = 0.0018), or be aged over 70 (*p* < 0.00001).

### 4.2. ICU Outcomes and Resuscitation Plan Status

A resuscitation plan was not a barrier to ICU admission with 460 (8.2%) patients with a plan passing through the ICU during their hospital stay. ICU admission was only slightly more common in patients without a resuscitation plan (9.7% vs. 8.2%, respectively; *p* = 0.002). ICU admission was, however, markedly more common in those with a delayed plan versus an early resuscitation plan (25% versus 6%; *p* < 0.00001).

As shown in Table 2, 1233/13,602 (9%) patients were admitted to the ICU during their inpatient stay, amongst whom 38% had a resuscitation plan completed at any time and 62% did not.

## 5. Discussion

### 5.1. Resuscitation Plan Completion and Mortality

The prevalence of resuscitation plan completion in our study was substantially higher (42%) than the previously reported 11.9% of No-CPR orders in Australian hospitals [14]. Patients more likely to have a resuscitation plan completed included those admitted under medical specialties, in-hours admissions, females, those aged over 70 years, those with LEP, and non-Indigenous Australians. Most resuscitation plans were completed early in admission (89%), and patients admitted under a medical team were more likely than surgical patients to have an early resuscitation plan. There was a trend of early completion in patients admitted in-hours, females, those over the age of 70 years, and non-Indigenous Australians. Language proficiency influenced the presence but not the timing of completion. Apart from the Indigenous Australian and LEP observations, these findings align with research about No-CPR orders from Japan [15], where, of those patients who survived to discharge, older patients, medical admissions, and females were most likely to have No-CPR orders in their health care record. Mori et al. [15] also demonstrated an association between early resuscitation plans and lower mortality rates when compared to delayed plans.

Of patients with a resuscitation plan, 9% died during their admission compared with 0.54% of those without a plan in place. This is not a surprising finding given that all clinical deterioration, critical illness, and anticipated death are triggers for proactive resuscitation planning. While the majority of patients who died had a resuscitation plan recorded (92%), 8% did not. These patients and their families were potentially denied the opportunity to participate in end-of-life care planning.

Patients overlooked for a resuscitation plan before death were more likely to be younger, male, and admitted under surgical specialties, and identify as Indigenous Australian. Patients with these characteristics were also more likely to have a delayed plan. Younger, surgical patients had a significantly lower crude mortality rate [Table 1] than their older, medical counterparts and this may have contributed to their being overlooked for resuscitation plans prior to death; the suggested triggers for initiating a discussion about resuscitation planning may not have been recognised.

Female patients were more likely to have a resuscitation plan, yet men have a shorter life expectancy and have a greater burden of disease than women [16]. Previous research has identified that men are more reluctant to talk about death or impending death than women [17], which may partly explain the lower rates of resuscitation plan completion in the male patient cohort.

Indigenous Australians exhibited significantly lower resuscitation plan completion rates and a higher likelihood of dying without a completed plan, despite similar in-hospital mortality rates compared to non-Indigenous Australians (Table 1). They were 5.5 times more likely to die without a resuscitation plan than non-Indigenous patients. There is a paucity of published data about qualitative and quantitative outcomes concerning Indigenous Australians and acute resuscitation planning in tertiary centres. The existing research exploring palliative care indicates lower-quality end-of-life care for Indigenous Australian patients [18,19]. This is potentially related to practitioners’ discomfort and misunderstanding when discussing death in an unfamiliar cultural context [20]. Moreover, Indigenous Australians experience a markedly decreased life expectancy compared to the general population [21] and access aged-care packages at a younger age [22]. This suggests that triggers for discussions are different for this population, and practitioners may not be comfortable recognising or discussing potential death during admission with Indigenous Australians.

Increased resuscitation plan completion in patients with LEP was unexpected. A previous qualitative study suggested that speaking a language other than English is perceived as a barrier to patients accessing health and services in the context of advance care resuscitation planning [23]. In a study of resuscitation status and end-of-life decision-making in the ICU in the United States, LEP patients had lower rates of No-CPR orders and advance directive completion [24]. As the LEP cohort had a higher mortality, this may partly explain the increased rate of end-of-life care planning documented in this group.

Out-of-hours admissions were associated with higher mortality rates, consistent with previous studies [25,26]; yet, resuscitation plan completion for patients who died remained similar regardless of admission timing. This suggests that end-of-life care discussions may occur more frequently with patients at a higher risk of deterioration, irrespective of admission time.

### 5.2. Resuscitation Plan Completion and ICU Admission

All patients admitted to the ICU warrant a resuscitation discussion within 48 h of their visit to promote patient autonomy and anticipate complications [27]. Despite the universally critical illness in this cohort, the prevalence of resuscitation plans among patients admitted to the ICU was less than the hospital-wide cohort, with 62% of ICU-admitted patients having no plan (Table 2). A US-based cohort study found a similar prevalence of resuscitation orders among patients admitted to the ICU—36.6% in their study [28]. Delayed resuscitation plan completion was associated with a higher likelihood of an ICU admission and increased mortality rates compared to early completion, emphasising the importance of timely end-of-life care planning.

Most resuscitation plans were completed for patients who neither died nor required ICU admission, indicating the potential use of these plans in lower-acuity settings to guide future care decisions. Recent studies found that the increased use of resuscitation plans was not associated with a significant change in the rates of ICU admissions or invasive interventions [29,30], which would support our finding of the significant use of resuscitation plans in a lower-acuity cohort.

## 6. Limitations

This was a single-centre, retrospective study that may not reflect findings in other Australian or international centres, and limits generalisability. These data cannot be extrapolated to obstetric, paediatric, intersex, mental health, nor elective admissions. COVID-19 was thought not to have a significant impact on this study. During the study period, there were 242 cases of COVID reported across the entire state of South Australia [31]. An analysis of demographic factors did not include potentially relevant variables such as socioeconomic status, rurality, educational background, specific languages, or cultural considerations that could influence resuscitation planning and outcomes. The study relied on electronic health records, which may contain inaccuracies or incomplete information. Resuscitation plans were not analysed for their specific content or quality, which likely has an impact on patient outcomes. Detailed patient preferences and family involvement in decision-making were not considered. Further research into the underlying causes for observed disparities would assist in creating solutions for the vulnerable demographic populations described.

## 7. Conclusions

At this centre, CPR and goals-of-care discussions are no longer an exclusively end-of-life occurrence, with 91% of patients with a documented resuscitation plan surviving to hospital discharge. Patients with early resuscitation plans had lower mortality and ICU admission rates compared to those with delayed plans, and only a minority of patients died without a plan in place. Overall, this suggests clinicians incorporate anticipatory planning into the early assessment and admission work-up for non-elective patients, in accordance with local and international guidelines.

Whilst total resuscitation plan completion rates were high, with most inpatients discussing goals of care before death, these discussions were occurring inconsistently across patient demographics. There were lower rates of early and total resuscitation plan completion for male patients, those under a surgical specialty, and those under 70 years old, and markedly lower rates for Indigenous Australians relative to their respective counterparts. Furthermore, 62% of patients admitted to the ICU did not have a resuscitation plan completed during their admission despite all these patients meeting the trigger criteria due to the severity of their illness.

Addressing disparities in resuscitation plan completion rates and ensuring equitable access to end-of-life care discussions are crucial steps towards improving patient-centred care and clinical outcomes. Further efforts are warranted to promote the consistent and timely engagement in resuscitation planning across all patient populations.

## Figures and Tables

**Table 1:** Resuscitation plan status in all admissions and those who died.

Variable Category	N Admissions (% of Total)	All Admissions		Patients Who Died			
		N with Plan Documented (%)	N Early Plan Documented (%)	N All (%)	N No Plan (%)	N Resuscitation Plan (%)	
						N Early Plan (%) ~	N Late Plan (%)
**Hospital-wide**	**13,718 (100)**	**5745 (42)**	**5107 (89)**	**540 (3.9)**	**43 (8)**	**497 (92)**	
						**405 (82)**	**92 (18)**
**Medical**	8245 (60)	4995 (60)	4492 (91)	473 (5.49)	25 (6)	428 (94)	
						358 (79)	70 (15)
**Surgical**	5473 (40)	790 (14) **	615 (78) **	87 (1.59) **	18 (21) **	69 (79)	
						47 (54) *	22 (25)
**In-hours**	**7009 (51)**	**3021 (43)**	**2712 (90)**	**250 (3.57)**	**22 (9)**	**282 (91)**	
						**186 (74)**	**42 (17)**
**Out-of-hours**	**6709 (49)**	2724 (41) *	2395 (88) *	290 (4.32) *	**21 (7)**	**269 (93)**	
						**219 (76)**	**50 (17)**
**Male**	7706 (56)	2985 (39)	2629 (88)	303 (3.92)	30 (10)	273 (90)	
						209 (69)	64 (21)
**Female**	6012 (44)	2760 (46) **	2478 (90) *	237 (3.94)	13 (5)	224 (95)	
						196 (83) *	28 (12)
**Age < 70 years**	**7644 (56)**	**1955 (26)**	**1704 (87)**	**165 (2.16)**	**26 (16)**	**139 (84)**	
						**90 (55)**	**49 (29)**
**Age ≥ 70 years**	**6074 (44)**	3790 (62) **	3403 (90) *	375 (6.17) **	17 (5) **	**358 (95)**	
						315 (84) **	**43 (11)**
**Primary language English**	12,112 (88)	4935 (41)	4392 (89)	446 (3.68)	33 (7)	413 (93)	
						336 (75)	77 (18)
**LEP**	1606 (12)	810 (50) **	715 (88)	94 (5.85) **	10 (11)	84 (89)	
						69 (73)	15 (16)
**Indigenous** **Australian**	**782 (6)**	**216 (28)**	**176 (81)**	**21 (2.69)**	**7 (33)**	**14 (67)**	
						**9 (43)**	**5 (24)**
**Non-indigenous**	**12,367 (90) ^†^**	5283 (43) **	4725 (89) *	**474 (3.83)**	29 (6) **	**445 (94)**	
						**366 (77)**	**79 (17)**

~ Statistical significance in this column compares the early resuscitation plan deaths with the late resuscitation plan deaths for each variable in the relevant cell above; i.e., bolded sections compare statistical significance between the binary categories (e.g. male and female) and vice versa. * *p* value < 0.05 when comparing with the variable immediately above (e.g., female versus male). ** *p* value < 0.001 when comparing with the variable immediately above (e.g., female versus male). ^†^ 569 (4%) patients were recorded as “Not stated” regarding their Indigenous status, and they were excluded from analysis for the Indigenous status variable category only.

**Table 2:** Timing of resuscitation plans and ICU admission.

ICU Admission Status	Number of Patients (Expressed as % Total Patients)	Number of Early Plan (Expressed as % of Total Patients in Category)	Number of Late Plan (Expressed as % of Total Patients in Category)	Number of No Plan (Expressed as % of Total Patients in Category)
Patients with 0 hour in ICU	12,369 (91)	4762 (38)	479 (4)	7128 (58)
Patients with >1 ICU hours ^†^	1233 (9)	311 (25)	156 (13)	766 (62)

^†^ 116 patients had no ICU length of stay recorded; these patients were excluded from analysis for this outcome only.

## Data Availability

The original data extracted are potentially re-identifiable due to linked hospital and personal demographic information. Reasonable requests for subsets of data will be considered if such information is not thought to risk patient confidentiality, in consultation with the Human Research Ethics Committee for Central Adelaide Local Health Network.

## Supplementary Materials

The following supporting information can be downloaded at: https://www.mdpi.com/article/10.3390/jcm13144098/s1, Additional File S1: 7-Step Pathway Resuscitation Plan.
