# Supplementary material for: A Retrospective Analysis of Characteristics Favouring In-Hospital Resuscitation Plan Completion, Their Timing, and Associated Outcomes
Source: J Clin Med. 2024 Jul 13;13(14):4098. doi: 10.3390/jcm13144098 (PMC11277889; doi:10.3390/jcm13144098)
Supplement: Supplementary file 1 [file jcm-13-04098-s001.zip › jcm-3075768-Additional File S1.pdf]

Tool 1: Resuscitation Plan - 7 Step Pathway Diagram

Government of South Australia
SA Health

STEP 1: TRIGGER

The clinical team caring for the patient should use standardised triggers to assess if a patient may be at end-of-life. If any of the of triggers are met, the clinician responsible for the patient should consider if an end-of-life clinical care plan is needed, the urgency for a plan, and readiness of patient/family to discuss issues.

Triggers:

1. The patient, family/carer, Substitute Decision-Maker, Person Responsible or members of the interdisciplinary team express concern or worry that the patient is dying and/or have unmet end-of-life care need.
2. Indicators are met using the Supportive and Palliative Care Indicators Tool (SPICT ™), a tool for identifying people at risk of deteriorating and dying (www.spict.org.uk/index.php).
3. The '**Surprise Question**': the clinician asks him or herself, "*Would I be surprised if this patient died in the next 12 months?* (and where the response is "**No**")".
4. A patient who has refused life-sustaining treatment in an Advance Care Directive (including in an Enduring Power of Guardianship, Medical Power of Attorney or Anticipatory Direction) or in an Advance Care Plan.
5. Observations triggering or are likely to trigger the activation of a Medical Emergency Response (MER).

STEP 2: ASSESSMENT

Obtain adequate clinical information to allow reasonable clinical decisions to be made, and to be the basis for discussions with the patient, Substitute Decision-Maker/ Person Responsible. Make an assessment about the capacity of the patient to participate in these discussions.

STEP 3: CONSULTATION

When the treating team has reached a clinical decision, sensitively, and clearly explain to the patient, Substitute Decision-Maker/ Person Responsible and others as indicated by the patient, the diagnosis, prognosis, treatment options and recommendations; and negotiate clear goals and intent for future treatment. Determine whether the patient has previously refused treatment. If the patient has lost capacity refer to Advance Care Directive/Advance Care Plan.

STEP 4: DOCUMENT THE CLINICAL CARE PLAN

Using the Resuscitation Plan form develop and document a realistic and practical clinical plan about resuscitation/life-sustaining measures, or treatment with a palliative approach, informed by the patient's wishes.

STEP 5: TRANSPARENCY AND COMMUNICATION

Explain the plan to the patient, Substitute Decision-Maker/ Person Responsible and others as indicated by the patient, in a consistent and compassionate way. Allow time for them to process the information, encourage questions and revisit as necessary to develop a shared understanding. If there is a dispute, then institute dispute resolution process as necessary.

STEP 6: IMPLEMENTATION

Take practical steps to implement the plan and revisit as necessary.

STEP 7: SUPPORT THE PATIENT, SUBSTITUTE DECISION-MAKER/ PERSON RESPONSIBLE AND FAMILY/CARERS

Throughout the process ensure practical, emotional and spiritual support is offered to the patient, Substitute Decision-Maker/ Person Responsible and family/carers including offering support and information after the patient has died.

For more information

SA Health
Safety and Quality Unit
www.sahealth.sa.gov.au/safetyandquality

For Official Use only: I1-A1

Interpreter

BY NC ND

www.ausgoal.gov.au/creative-commons

© Department for Health and Ageing, Government of South Australia.
All rights reserved. FIS: 16000.5 April 2016

Government of South Australia
SA Health
